# Phenotypic and Proteomic Analysis of the *Aspergillus fumigatus* Δ*PrtT*, Δ*XprG* and Δ*XprG*/Δ*PrtT* Protease-Deficient Mutants

**DOI:** 10.3389/fmicb.2017.02490

**Published:** 2017-12-12

**Authors:** Einav Shemesh, Benjamin Hanf, Shelly Hagag, Shani Attias, Yana Shadkchan, Boris Fichtman, Amnon Harel, Thomas Krüger, Axel A. Brakhage, Olaf Kniemeyer, Nir Osherov

**Affiliations:** ^1^Aspergillus and Antifungal Research Laboratory, Department of Clinical Microbiology and Immunology, Sackler School of Medicine, Tel Aviv University, Tel Aviv, Israel; ^2^Leibniz Institute for Natural Product Research and Infection Biology, Hans Knöll Institute (HKI), Jena, Germany; ^3^Institute of Microbiology, Friedrich Schiller University, Jena, Germany; ^4^Faculty of Medicine in the Galilee, Bar-Ilan University, Safed, Israel

**Keywords:** *Aspergillus fumigatus*, protease secretion, transcription factor, proteomics, virulence

## Abstract

*Aspergillus fumigatus* is the most common mold species to cause disease in immunocompromised patients. Infection usually begins when its spores (conidia) are inhaled into the airways, where they germinate, forming hyphae that penetrate and destroy the lungs and disseminate to other organs, leading to high mortality. The ability of hyphae to penetrate the pulmonary epithelium is a key step in the infectious process. *A. fumigatus* produces extracellular proteases that are thought to enhance penetration by degrading host structural barriers. This study explores the role of the *A. fumigatus* transcription factor XprG in controlling secreted proteolytic activity and fungal virulence. We deleted *xprG*, alone and in combination with *prtT*, a transcription factor previously shown to regulate extracellular proteolysis. *xprG* deletion resulted in abnormal conidiogenesis and formation of lighter colored, more fragile conidia and a moderate reduction in the ability of culture filtrates (CFs) to degrade substrate proteins. Deletion of both *xprG* and *prtT* resulted in an additive reduction, generating a mutant strain producing CF with almost no ability to degrade substrate proteins. Detailed proteomic analysis identified numerous secreted proteases regulated by XprG and PrtT, alone and in combination. Interestingly, proteomics also identified reduced levels of secreted cell wall modifying enzymes (glucanases, chitinases) and allergens following deletion of these genes, suggesting they target additional cellular processes. Surprisingly, despite the major alteration in the secretome of the *xprG/prtT* null mutant, including two to fivefold reductions in the level of 24 proteases, 18 glucanases, 6 chitinases, and 19 allergens, it retained wild-type virulence in murine systemic and pulmonary models of infection. This study highlights the extreme adaptability of *A. fumigatus* during infection based on extensive gene redundancy.

## Introduction

*Aspergillus fumigatus* is a common saprophytic mold which produces abundant microscopic conidia (2–4 micrometers) that can be inhaled into the pulmonary alveoli to cause a variety of pathological conditions (Kwon-Chung and Sugui, 2013). In the context of the immunocompromised patient, the infection, termed Invasive Pulmonary Aspergillosis (IPA), is life-threatening and severe (Kosmidis and Denning, 2015). The neutropenic status of these patients culminates in their inability to destroy inhaled conidia resulting in fungal growth and penetration through the pulmonary epithelium into the blood stream (Latge, 2001).

The success of *A. fumigatus* as a pathogen is not a result of direct adaptation to the host. Rather it should be viewed as an accidental interaction between a common and hardy environmental mold and a weakened host. *A. fumigatus* survives in the compromised host due to a chance combination of pre-existing capabilities. They include the abundant release of small conidia protected by a non-immunogenic layer of hydrophobins and oxygen-radical quenching pigments. Growing hyphae effectively endure oxidative stress and hypoxia, efficiently collect scarce iron and secrete toxins that further depress host immune function and proteases that degrade host tissue (Abad et al., 2010; Kwon-Chung and Sugui, 2013). In human A549 alveolar epithelial cells, culture filtrates (CFs) of *A. fumigatus* can disrupt the actin cytoskeleton, activate NFκB signaling and induce the production of proinflammatory cytokines. These cellular events can be prevented by addition of serine protease inhibitors to the secreted CF, implying that they are directly dependent on secreted fungal proteases (Kogan et al., 2004; Sharon et al., 2011). *A. fumigatus*-secreted proteases and gliotoxin also induce platelet activation that may serve as a mechanism for activating the immune defenses and inducing inflammation (Speth et al., 2013). Additionally, the secreted *A. fumigatus* alkaline protease Alp1 cleaves the complement components C3, C4, and C5 that contribute to evasion from the host immune response (Behnsen et al., 2010). Alp1 is also a major allergen (Aspf 13) and promotes airway hyper-responsiveness and bronchoconstriction in asthma (Balenga et al., 2015).

In previous work we have identified the transcription factor PrtT, a positive regulator of secreted proteases in *A. fumigatus*. Deletion of *prtT* results in greatly reduced secreted protease activity and a reduction in the transcription of secreted proteases. Δ*prtT* CF showed reduced killing of A549 lung alveolar cells and erythrocyte lysis (Bergmann et al., 2009; Sharon et al., 2009, 2011). However, the Δ*prtT* strain showed wild-type virulence in infected neutropenic mice suggesting that perhaps residual protease activity was sufficient to enable virulence in this setting (Bergmann et al., 2009; Sharon et al., 2009).

In the related mold *A. nidulans*, that lacks a *prtT* homolog, the transcription factor XprG regulates extracellular protease production in response to nutrient stress (Katz et al., 2013, 2015; Katz and Cooper, 2015). Deletion of *A. nidulans xprG* resulted in complete loss of halo formation on skimmed milk (SM) agar plates and an inability to grow on medium containing BSA as sole carbon or nitrogen source. XprG is a member of the p53-like transcription factors, also known as the *NDT80/PhoG*-like family. *NDT80* transcription factors are found in animals, fungi, and amoeba. Fungal *NDT80* genes were studied in detail in *Saccharomyces cerevisiae* (Pak and Segall, 2002), *Neurospora crassa* (Hutchison and Glass, 2010) *Candida albicans* (Chen et al., 2004; Sellam et al., 2009, 2010) and *A. nidulans* (Katz et al., 2013, 2015; Katz and Cooper, 2015). The consensus is that in response to nutrient stress and deprivation they activate specific target genes such as proteases, phosphatases, secondary metabolites, and genes involved in meiosis (itself a stress response initiated by starvation) and autolysis.

We hypothesized that XprG also co-regulates protease production in *A. fumigatus* and that deletion of both *prtT* and *xprG* would completely abolish secreted protease activity, reducing fungal virulence. To test this hypothesis we prepared *A. fumigatus* strains deleted in *prtT*, *xprG* alone and in combination. The effects of these mutations *in vitro* and during infection *in vivo* are described.

## Materials and Methods

### Strains and Culture Conditions

The strains used in this study are detailed in **Table 1**. *A. fumigatus* conidia were harvested in 0.2% (*vol/vol*) Tween 20, resuspended in double-distilled water (DDW) and counted with a hemocytometer. For continuous growth, *A. fumigatus* strains were grown on YAG medium, that consists of 0.5% (*wt/vol*) yeast extract, 1% (*wt/vol*) glucose, and 10 mM MgCl_2_, supplemented with trace elements, vitamins, and 1.5% (*wt/vol*) agar when needed (Bainbridge, 1971). SM medium consisted of 1% (*wt/vol*) glucose, 1% (*wt/vol*) SM (Difco, Livonia, MI, United States), 0.1% (*wt/vol*) Casamino Acids (Difco), 7 mM KCl, 2 mM MgSO_4_ and 50 mM Na_2_HPO_4_-NaH_2_PO_4_ buffer (pH 5.3), supplemented with vitamins, trace elements and 1.5% agar when needed. NaNO_3_-depleted *Aspergillus* minimal medium (Weidner et al., 1998) was used for collagen medium with 0.1% (*wt/vol*) yeast extract, 0.5% (*wt/vol*) glucose and collagen as sole carbon and nitrogen source. Peptone medium contained 1% (*wt/vol*) glucose, 0.4% peptone (Difco), 7 mM KCl, 2 mM MgSO_4_ and 50 mM Na_2_HPO_4_-NaH_2_PO_4_ buffer (pH 5.3), supplemented with vitamins and trace elements. Genetically modified organisms and pathogens used in this study were maintained in accordance with TAU Institutional Policies.

**Table 1:** Strains used in this study.

Strain	Genotype	Source
*KU80 (ΔakuB)*	CEA17, AFUA_2G02620::*pyrG*	da Silva Ferreira et al., 2006
Δ*PrtT*	AFUA_4G10120::*hph*	This work
Δ*XprG*	AFUA_8G04050:: *ptrA*	This work
Δ*XprG/ΔPrtT*	AFUA_8G04050:: *ptrA*;	This work
	AFUA_4G10120::*hph*	
*PrtT KI*	AFUA_4G10120::*hph*;	This work
	AFUA_4G10120-*phl*	
*XprG KI*	AFUA_8G04050:: *ptrA*	This work
	AFUA_8G04050-hph	
Δ*XprG/PrtT KI*	AFUA_8G04050:: *ptrA*;	This work
	AFUA_4G10120::*hph*	
	AFUA_4G10120-*phl*	


### Generation and Verification of *A. fumigatus* Mutant Strains

All strains were prepared in the *Ku80* null background, strain *AkuB^KU*80*^* (da Silva Ferreira et al., 2006). Full details of the construction and verification of the strains shown in **Table 1**, including a list of the primers used (Supplementary Table S1), are provided in the Supplementary Data Section.

### Conidial Stability in Detergent Storage

Freshly harvested *A. fumigatus* conidia (10^7^/ml) were suspended in DDW +0.5% (*vol/vol*) Tween 20 at 37°C. At different time-points, aliquots were diluted, plated on YAG plates and the number of colonies counted.

### Conidial Disruption by Glass Beads

Freshly harvested *A. fumigatus* conidia (5 × 10^7^/ml) were suspended in 0.5 ml DDW +0.1% Tween 20 and mixed with 0.5 ml (packed volume) of acid washed glass beads, 150–212 μm (Sigma–Aldrich Corp., St. Louis, MO, United States). They were then vortexed on medium strength for up to 10 min. At each time point a sample was taken, diluted and plated on YAG plates. The plates were incubated at 37°C for 24–36 h, colonies were counted and survival rates were calculated as the percentage of viable spores.

### Analysis of Fungal Enzymatic Activity

Proteolytic activity on solid medium was assessed by spotting conidia on SM plates containing 0.1% Tween 20. The colonies were grown for 48 h at 37°C, then transferred to room temperature for another 48 h and subsequently photographed. Supernatants were collected from *Aspergillus* cultures grown in liquid SM for 48 h at 37°C. Azocasein (Sigma) was dissolved at a concentration of 5 mg/ml in assay buffer containing 50 mM Tris (pH 7.5), 0.2 M NaCl, 5 mM CaCl_2_ and 0.05% Triton X-100 as previously described (Kogan et al., 2004). The azocasein solution (400 μl) was mixed with 100 μl portions of supernatants from *Aspergillus* cultures and incubated by shaking for 90 min at 37°C. The reactions were stopped by adding of 150 μl 12% (*vol/vol*) trichloroacetic acid, and the reaction mixtures were allowed to stand at room temperature for 30 min. Tubes were then centrifuged for 3 min at 8,000 *g*, and 100 μl of each supernatant was added to 100 μl of 1 M NaOH. The absorbance of released azo dye at 436 nm was determined with a spectrophotometer. Proteolytic activity on bovine serum albumin (BSA) was measured by growing *A. fumigatus* in 24-well plates with 1 ml/well liquid peptone medium containing 0.1% BSA, respectively, for 24–72 h at 37°C. Supernatants were boiled in sample buffer and run on an 8% SDS-PAGE gel followed by Coomassie staining to visualize the proteins.

### Scanning Electron Microscopy (SEM) Analysis

*Aspergillus fumigatus* wild-type and mutant strains were grown for 72 h at 37°C on YAG agar plates. Fixation and processing of samples for SEM was performed as described earlier (Fichtman et al., 2014; Hover et al., 2016). Briefly, small areas of conidiating mycelium were carefully excised from the zone of interaction and vapor fixed with 8% (*vol/vol*) paraformaldehyde and 4% (*vol/vol*) glutaraldehyde dissolved in water for 1 h in a closed chamber. Secondary vapor fixation was then carried out in an aqueous solution of 2% (*wt/vol*) osmium tetroxide for 1 h. The samples were submerged for 10 min in DDW and then dehydrated (10 min, twice for each step) under a series of ethanol concentrations (7.5, 15, 30, 50, 70, 90, 95, and 100%). Next, samples underwent critical point drying (CPD) using a K850 CPD dryer (Quorum Technologies, United Kingdom). Coating was done with 3 nm iridium using a Q150T coater (Quorum Technologies, United Kingdom). Samples were imaged with a Merlin scanning electron microscope (Zeiss, Germany).

### LC-MS/MS Analysis and Identification of Secreted Proteins

*Aspergillus fumigatus* strains at a concentration of 10^6^ condia/ml were grown in liquid collagen medium for 72 h at 37°C. Total protein was TCA-precipitated from the CFs, digested with trypsin, iTRAQ-labeled and analyzed by liquid chromatography-tandem mass spectrometry (LC-MS/MS) as detailed in the Supplementary Data.

### Murine Models for Invasive Aspergillosis

For the cyclophosphamide/cortisone acetate neutropenic model (Ejzykowicz et al., 2009), 6-week-old female ICR mice were injected intraperitoneally with cyclophosphamide (150 mg/kg in PBS) at 3 days prior to infection, on the day of infection and at 3 days post-infection. Cortisone acetate (150 mg/kg PBS with 0.1% Tween 20) was injected subcutaneously at 3 days prior to conidial infection.

For intranasal infection, mice were anesthetized by intraperitoneal injection of a solution of 250 μl xylazine (VMD, Arendonk, Belgium) and ketamine (Imalgene, Fort Dodge, IA, United States) at a concentration of 1.0 and 10 mg/ml, respectively (dissolved in PBS). Following anesthesia, the mice were inoculated intranasally with 2.5 × 10^5^ (intranasal model) or intravenously (IV) through the tail vein (IV model) with freshly harvested conidia of *AkuB^KU*80*^*, Δ*XprG*, or Δ*XprG/*Δ*PrtT* in PBS +0.1% (*vol/vol*) Tween 20. The inoculum was verified by quantitative culture. The animals were monitored for survival for up to 18 days. For infection of immunocompetent mice, 6-week-old female ICR mice were inoculated intranasally with 1 × 10^7^ conidia and sacrificed 24 h or 48 h later. Excised lungs were ground and plated on YAG agar plates for CFU enumeration. Histological analysis was performed with Gomori methenamine silver stain (GMS, stains fungal elements black) or haematoxylin and eosin stain (H&E, stains host-cell nuclei purple, cytosol pink). Statistical analysis of mouse survival was performed with GraphPad Prism 4 software (GraphPad Software, San Diego, CA, United States). Animal studies were authorized by the Tel Aviv University Animal Welfare Committee according to the Israel Ministry of Health guidelines and carried out in accordance with Tel Aviv University institutional policies.

## Results

### Generation of *A. fumigatus prtT*, *xprG*, *xprG/prtT* Null Mutants and Reconstituted Strains

A single *A. fumigatus xprG* ortholog (AFUA_8G04050) was identified by amino-acid similarity to *A. nidulans* XprG (70% identity). It is predicted to be 1752 nucleotides long and to contain one intron. *A. fumigatus* XprG encodes a protein 583 amino acids in length, containing a predicted *NDT80*/*PhoG* like DNA-binding domain (amino-acid residues 156–327) and a MAP65/ASE1 domain (amino acid residues 336–505) shared by microtubule-binding proteins. The similarity to *NDT80* family genes *vib-1* from *N. crassa* and *S. cerevisiae ndt80* is limited to the *PhoG* like DNA-binding domain. To analyze the function of XprG and its interaction with PrtT in *A. fumigatus*, we prepared Δ*PrtT*, Δ*XprG*, Δ*XprG/*Δ*PrtT* null mutants and corresponding reconstituted strains *PrtT-KI*, *XprG-KI*, and *PrtT-KI/*Δ*XprG* by transformation with a circular plasmid containing the *PrtT* gene and the phleomycin resistance cassete for selection (see Supplementary Data for full details about strain construction and verification).

### Abnormal Conidiogenesis in the Δ*Xprg* and Δ*XprG/*Δ*PrtT A. fumigatus* Mutants

**FIGURE 1:**
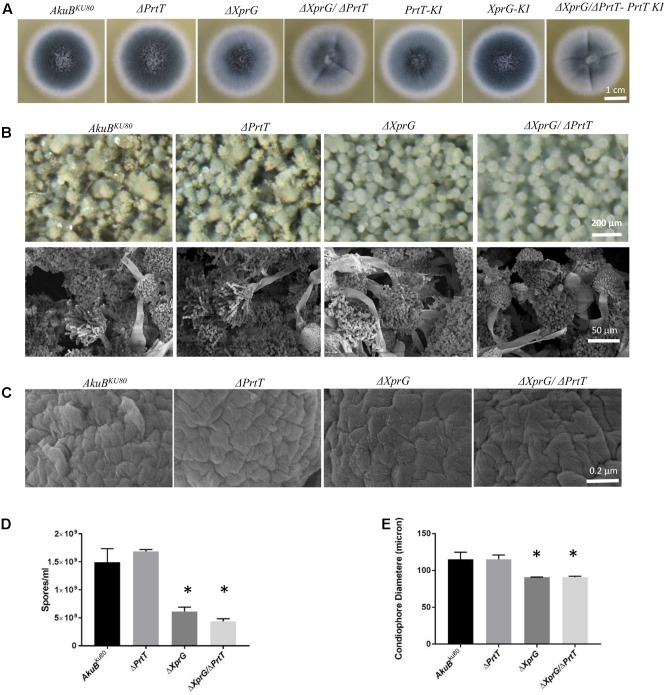
Deletion of *xprG* and *xprG*/*prtT* leads to abnormal conidiogenesis. Phenotypic analysis of the Δ*XprG* and Δ*XprG/*Δ*PrtT* strains compared to the wild-type *AkuB^KU*80*^* and Δ*PrtT* strains shows that they produce **(A)** lighter colored conidia when growing on YAG agar plates **(B)** conidiophores with reduced diameter and shorter spore chains (Light microscopy and SEM) **(C)** conidia with a normal rodlet outer layer (SEM, high magnification), **(D)** a significantly reduced number of conidia (^∗^*P* < 0.005). **(E)** Quantification of reduction in conidiophore diameter (based on the diameter of 50 conidiophores per strain) (^∗^*P* < 0.02).

The aforementioned null and reconstituted strains were point inoculated on YAG agar plates and examined for differences in morphology after growth for 72 h at 37°C. Results show that the Δ*XprG*, Δ*XprG/*Δ*PrtT* null strains produced lighter colored conidia as compared to the *AkuB^KU*80*^*, Δ*PrtT*, and *XprG-KI* strains (**Figure 1A**). Examination of the conidiophore structure by light microscopy and SEM revealed that the Δ*XprG* and Δ*XprG/*Δ*PrtT* strains produced significantly smaller, more compact conidiophores (*P* < 0.02) with shorter conidial chains (**Figures 1B,E**). The conidial hydrophobin outer rodlet layer remained intact and unchanged (**Figure 1C**) and the surface hydrophobicity of the mutant colonies, based on their ability to exclude water, was unchanged (not shown). Reflecting the shorter conidial chains, the number of conidia produced by the Δ*XprG* and Δ*XprG/*Δ*PrtT* strains was significantly reduced three–fourfold per plate (*P* < 0.05) compared to *AkuB^KU*80*^* and Δ*PrtT* (**Figure 1D**). Radial growth rates of the Δ*PrtT*, Δ*XprG*, Δ*XprG/*Δ*PrtT* strains were similar to that of *AkuB^KU*80*^* at both 37 and 48°C (Data not shown). The lighter conidial color of the *PrtT-KI* complemented strain compared to the deleted strain (**Figure 1A**) is unexpected and may be due to multiple integration of the complementing plasmid.

### The Conidia Produced by the Δ*Xprg* and Δ*XprG/*Δ*PrtT A. fumigatus* Mutants Are More Fragile

**FIGURE 2:**
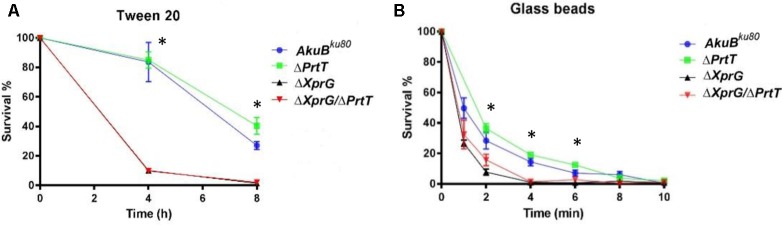
The Δ*XprG* and Δ*XprG/*Δ*PrtT* mutants produce conidia that are more sensitive to chemical and mechanical stress. **(A)** 10^3^ conidia/ml were suspended in 0.5% Tween 20 for the time indicated, plated and the number of colonies counted in comparison to controls suspended in DDW alone. ^∗^*P* < 0.001 *AkuB^ku*80*^* vs. Δ*XprG* and Δ*XprG/ΔPrtT* mutants. **(B)** 5 × 10^7^ conidia/ml were agitated in the presence of acid-washed glass beads for the time indicated, plated and the number of colonies counted in comparison to untreated controls. ^∗^*P* < 0.05 *AkuB^ku*80*^* vs. Δ*XprG* and Δ*XprG/*Δ*PrtT* mutants.

We evaluated the ability of the mutant conidia to withstand osmotic or detergent disruption by measuring their survival in water or in water containing Tween 20 detergent. Conidia from the Δ*XprG* and Δ*XprG/*Δ*PrtT* strains exhibited similar increased susceptibility to the detergent, losing >90% viability after 4 h as compared to loss of only <20% viability in the *AkuB^KU*80*^* and Δ*PrtT* strains (**Figure 2A**). There were no differences in conidial stability during storage in DDW, suggesting the mutants are not osmotically sensitive (data not shown). The ability of the mutant conidia to withstand physical disruption was assessed by subjecting them to agitation in the presence of glass beads. Conidia from the Δ*XprG* and Δ*XprG/*Δ*PrtT* strains exhibited increased susceptibility to glass-bead agitation, losing all viability after 4 min agitation, compared to 10 min for the *AkuB^KU*80*^* and Δ*PrtT* strains (**Figure 2B**).

Taken together these results show that deletion of *xprG* in the *AkuB^KU*80*^* and Δ*PrtT* strains resulted in the production of smaller conidiophores containing less and more fragile spores.

### Deletion of *xprG* and *prtT* in *A. fumigatus* Results in an Additive Reduction in the Ability to Degrade Casein and Albumin

**FIGURE 3:**
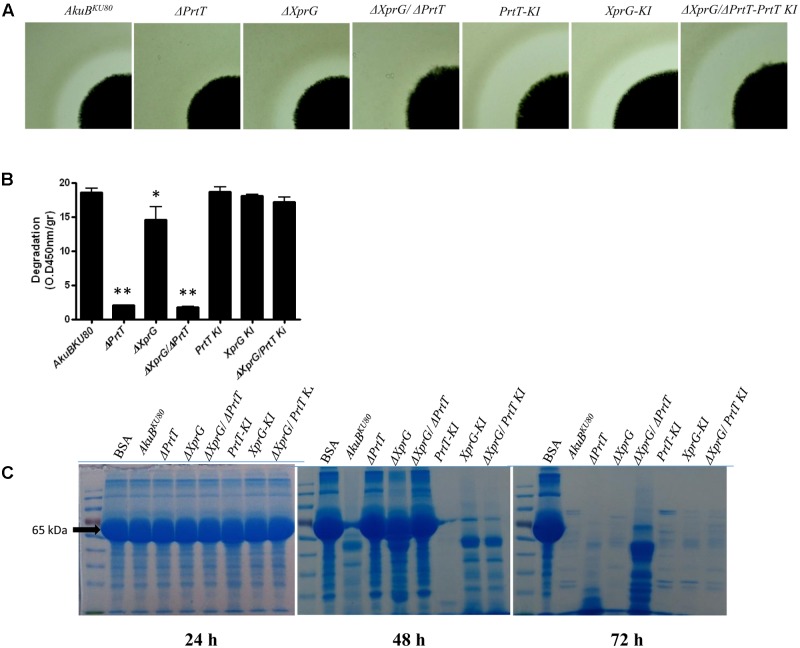
Proteolytic activity following deletion of *prtT* and *xprG*. Proteolytic activity of the fungal strains was assessed by **(A)** Halo formation on SM agar plates containing 0.1% Tween 20 following 48 h of growth at 37°C followed by another 48 h at room temperature. **(B)** Degradation of azocasein by *A. fumigatus* culture filtrates (CFs). The absorbance of the released azo dye was measured spectrophotometrically at 450 nm. (^∗^*P* < 0.05, ^∗∗^*P* < 0.0001). **(C)** BSA proteolysis by *A. fumigatus* CFs followed by SDS-PAGE analysis of the cleaved substrate. BSA (65 kDa, arrow) and other proteins were stained by Coomassie.

To investigate the effect of *xprG* deletion on secreted protease activity, we point inoculated the strains on SM agar plates and visually determined the formation of a proteolytic halo due to casein degradation around the fungal colony. As we have previously shown (Sharon et al., 2009), deletion of *prtT* resulted in the formation of a very weak halo compared to the control strain *AkuB^KU*80*^*, whereas deletion of *xprG* (Δ*XprG*) led to a partially reduced halo. Deletion of both *xprG* and *prtT* (Δ*XprG/*Δ*PrtT)* completely abolished the formation of a proteolytic halo (**Figure 3A**) and reduced azocasein degradation by over 90% (**Figure 3B**). Halo formation was restored in the reconstituted strain *XprG-KI* and slightly increased in the *PrtT-KI* and *PrtT-KI/*Δ*XprG* strains. This increase, however, was not seen in the azocasein or BSA degradation assays described below. We used a more sensitive SDS-PAGE-based BSA degradation assay to better differentiate between the proteolytic activities of the Δ*XprG*,Δ*PrtT* and Δ*XprG/*Δ*PrtT* mutants (**Figure 3C**). Strains were grown for 24–72 h on peptone medium containing 0.1% BSA. The supernatant was separated on an SDS-PAGE gel followed by Coomassie staining to visualize the degradation of the BSA protein. The control *AkuB^KU*80*^* and reconstituted strains substantially degraded the BSA substrate protein after 48 h of incubation, whereas the Δ*PrtT*, Δ*XprG*, and Δ*XprG/*Δ*PrtT* strains did not (**Figure 3C**). After 72 h of incubation, all strains except Δ*XprG/*Δ*PrtT* had completely degraded the BSA. Taken together, our findings indicate that deletion of both *prtT* and *xprG* results in an additive reduction in the ability to degrade casein or albumin. Previous analyses using protease inhibitors and deletion mutants have demonstrated that secreted *A. fumigatus* serine proteases, and in particular Alp1, are primarily responsible for the degradation of these substrates (Jaton-Ogay et al., 1994; Tomee et al., 1997; Kogan et al., 2004).

### Proteomic Analysis of the Δ*PrtT*, Δ*XprG*, and Δ*XprG/*Δ*PrtT* Secretomes

To identify the repertoire of proteins secreted by the Δ*PrtT*, Δ*XprG*, and Δ*XprG/*Δ*PrtT* mutants compared to the control *AkuB^KU*80*^* strain, conidia were cultured in liquid MM containing collagen for 72 h at 37°C with shaking. Supernatants were TCA-precipitated, treated after Yeung and Stanley (2009), followed by a Wessel-Flügge precipitation, labeled with iTRAQ 4-plex isobaric labeling for quantitative proteomics approach and analyzed by LC-MS/MS. A total of 938 proteins were identified in the fungal supernatants, 274 of which were predicted to be secreted by *in silico* analysis (secretion signal found by signalP for 236 proteins and targetP for 274 proteins) (Supplementary Table S2).

Cell lysis cannot be completely excluded during cultivation and sample preparation. It is therefore possible that a proportion of the secretome consisted of proteins without a secretion signal, which had been released by cell lysis. However, the comparison of the abundance (based on normalized PSM values) of typical intracellular, non-secreted proteins like actin (*Afu6g04740*) or tubulin (*Afu1g02550*) with the abundance of a secreted protein like lap2 (*Afu3g00650*) revealed a depletion by a factor between 12 and 106. In addition, fungi are able to release typical intracellular proteins via extracellular vesicles to the extracellular space (Joffe et al., 2016). It can be therefore assumed that the contamination of the secretome by cell lysis was relatively low.

**FIGURE 4:**
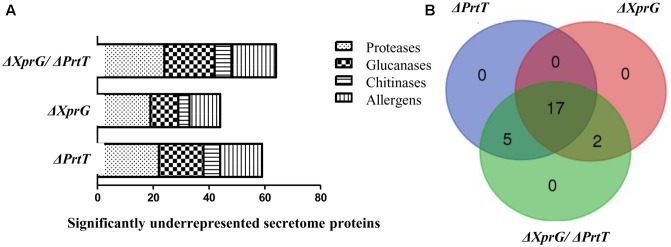
Effect of the deletion of *prtT*, *xprG* and *xprG*/*prtT* on the secretome of *A. fumigatus*. Deletion of *prtT*, *xprG* and *xprG*/*prtT* **(A)** reduces the secretion not only of proteases but also of glucanases and chitinases participating in cell wall biosynthesis and of numerous allergenic proteins. **(B)** Venn diagram illustrating the degree of overlap in the secretome datasets of the mutant strains.

Deletion of *prtT* resulted in strongly reduced secretion of 22 proteases, including Lap1, Lap2, Cp1, Cp3 Alp1, Alp2, and Mep (**Figure 4** and Supplementary Table S3). Deletion of *xprG* resulted in reductions in the secretion of 19 proteases, including most strongly Lap1, Sxa2, DppV, DppIV, Mep, and SedC. Notably, several proteases that were strongly reduced in the Δ*PrtT* mutant were only slightly reduced (Pep2, DapB, Cps1, Cp1, Cp3) or not reduced (CtsD, Ape3, Lap2, Alp1, Alp2) in the Δ*XprG* strain (**Figure 4** and Supplementary Table S3). This provides a good explanation for the higher secreted protease activity of the Δ*XprG* vs. the Δ*PrtT* mutant, especially the lack of reduction in the major neutral serine protease Alp1 (Behnsen et al., 2010). Deletion of both *prtT* and *xprG* resulted in the strongest reductions in the secretion of 24 proteases comprising the entire joint dataset of the Δ*PrtT* and Δ*XprG* mutants, including 14 serine proteases, 7 metalloproteases and 3 aspartic proteases (**Figure 4** and Supplementary Table S3). Interestingly, in all three mutant strains, the secretion of numerous glucanases, chitinases, and allergens was also reduced, indicating that these transcription factors not only regulate the expression of secreted proteases but also of cell wall enzymes and antigenic proteins (Supplementary Table S3). The significance of these results will be explained in the discussion. No proteins showed increased secretion in the mutants vs. the control *AkuB^KU*80*^* strain.

### The Δ*XprG* and Δ*XprG/*Δ*PrtT* Strains Exhibit Normal Virulence in Infected Mice

**FIGURE 5:**
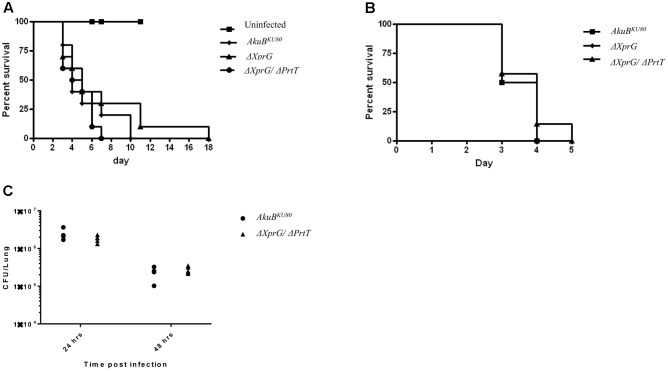
Deletion of *xprG* and *xprG*/*prtT*, does not affect virulence in a neutropenic murine model of **(A)** pulmonary aspergillosis, **(B)** disseminated aspergillosis and **(C)** pulmonary fungal load in immunocompetent mice. Cortisone acetate plus cyclophosphamide (neutropenia model) were used to immunocompromise the infected mice **(A,B)**. Survival curves are shown for mice infected **(A)** intranasally with an inoculum of 2.5 × 10^5^ conidia per mouse (*n* = 10 mice/group) and **(B)** intravenously via the lateral tail vein with an inoculum of 2.5 × 10^5^ conidia per mouse (*n* = 7 mice/group). These experiments were repeated twice with similar results. **(C)** Immunocompetent mice were infected intranasally with an inoculum of 1 × 10^7^ conidia per mouse (*n* = 5 mice/group) and total lung fungal load (CFU/lung) assessed 24 and 48 h post-infection. (Additional histological analysis is presented in the Supplements- Supplementary Figure S6).

Previous studies have shown that deletion of *prtT* in *A. fumigatus* does not affect virulence in lung-infected neutropenic mice (Bergmann et al., 2009; Sharon et al., 2009). We hypothesized that the almost complete lack of secreted protease activity in the Δ*XprG/*Δ*PrtT* strain would result in a noticeable reduction in virulence. To test this, we compared the virulence of the control *AkuB^KU*80*^*, Δ*XprG* and Δ*XprG/*Δ*PrtT* strains in two mouse models of invasive aspergillosis. Both models reproduce profound neutropenia (as found, for example following chemotherapy in leukemic patients), by immunosuppressing the mice with a combination of cortisone acetate and cyclophosphamide. In the first model, the mice were infected intranasally with freshly harvested conidia. In the second model reproducing disseminated infection, mice were infected IV. The number of live mice in each group was recorded daily throughout the experiment. The Δ*PrtT* strain was not included as we have previously shown it to be fully virulent in these models (Sharon et al., 2009). **Figure 5** shows survival curves obtained during the course of the experiment for the lung (**Figure 5A**) and disseminated (**Figure 5B**) models. No significant differences in virulence were found for the Δ*XprG* and Δ*XprG/*Δ*PrtT* mutants compared to the control *AkuB^KU*80*^* strain (*P*-value > 0.3). Next, we analyzed the response of immunocompetent mice to intranasal pulmonary infection (10^7^ conidia/mouse) with Δ*XprG/*Δ*PrtT* and control *AkuB^KU*80*^* strains. Outputs included lung CFU (**Figure 5C**) and histology (Supplementary Figure S6), 24 and 48 h following infection. There was no significant difference in lung fungal load (*P*-value > 0.2) between the Δ*XprG/*Δ*PrtT* and control *AkuB^KU*80*^* strains (**Figure 5C**). Histology revealed abundant conidia in the bronchi of both mutant and control strains 24 h after infection (CMS stain, right panel, arrows) accompanied by a considerable influx of red blood cells and lymphocytes (H&E stain, left panel) (Supplementary Figure S6). As expected in immunocompetent mice, the number of conidia strongly decreased 48 h after infection, as did the number of red blood cells and lymphocytes (Supplementary Figure S6). Taken together, these results suggest that immunocompetent mice mount a similar and effective immune response to both Δ*XprG/*Δ*PrtT* and control *AkuB^KU*80*^* infection.

## Discussion

This study describes the disruption of the putative transcriptional activator XprG in *A. fumigatus*, alone and in combination with the previously described transcription factor PrtT. We chose to study XprG in *A. fumigatus* because (i) it regulates protease expression in *A. nidulans* and we reasoned it may also do so, in combination with PrtT, in *A. fumigatus*, (ii) regulation of protease expression by combinations of transcription factors in pathogenic filamentous fungi is poorly understood and (iii) we hypothesized that deletion of two transcriptional regulators controlling the expression of multiple proteases could shed more light on the involvement of secreted proteases in the pathogenesis of *A. fumigatus*.

We gained several important insights from this study. First, we found considerable divergence in the role of XprG in *A. nidulans* and *A. fumigatus*. In *A. nidulans* that lacks a PrtT homolog XprG is the major activator of secreted protease activity under starvation. In *A. fumigatus*, this function is carried out by PrtT, with XprG performing a secondary role. Increasing functional divergence is found in the *N. crassa* XprG homolog vib-1 – in addition to controlling conidial pigmentation and increasing extracellular protease activity it also controls protoperithecial sexual development (Hutchison and Glass, 2010). Further functional separation has occurred in *S. cerevisiae* ndt80 (Pak and Segall, 2002) that controls meiosis and *C. albicans* CaNdt80 (Chen et al., 2004; Sellam et al., 2009, 2010), that regulates azole resistance, hyphal growth and virulence. The basis for this diversity likely stems from the extreme sequence divergence outside of the conserved *NDT80*/*PhoG* like DNA-binding domain.

We also found evidence for overlap in ndt80/vib-1/XprG function. As previously shown in *A. nidulans xprG* and *N. crassa vib-1* null mutants, deletion of *A. fumigatus xprG* resulted in the formation of pale conidia, suggesting a role in melanin biosynthesis. We further showed that in *A. fumigatus*, conidiogenesis is impaired and the resulting conidia are weaker and more prone to chemical and physical disruption. Most interestingly, we show here that *A. fumigatus* XprG and PrtT activate expression of glucanases, chitinases and numerous allergens. *C. albicans* CaNdt80 also activates genes encoding cell wall components including expression of chitinase Cht3p and cell wall glucosidase Sun41p that are essential for the completion of cell separation (Sellam et al., 2010). However, despite reduced levels of numerous secreted glucanases and chitinases in *A. fumigatus* Δ*XprG*, we found no obvious evidence for cell wall alterations by either SEM, transmission electron microscopy (TEM) or cell wall inhibitor analysis (data not shown). CaNdt80 also activates CDR1 efflux pump and ergosterol biosynthesis and its deletion results in azole sensitivity. Nevertheless, deletion of *A. fumigatus xprG* did not increase azole sensitivity (data not shown) suggesting that it does not affect these targets.

Our analysis of XprG and PrtT showed that both genes are not essential for maintaining virulence. Considering that the double-mutant has almost no detectable secreted protease activity *in vitro* and exhibits strong (two–fivefold) reductions in 24 secreted proteases [including all 5 proteases that are activated during *in vivo* infection (McDonagh et al., 2008)] as well as 18 glucanases and 6 chitinases, the unaffected virulence in the mouse model is unexpected. The *A. fumigatus* genome encodes approximately 50 putative secreted proteases, and some may be alternatively activated *in vivo*, compensating for the reductions seen in Δ*PrtT/*Δ*XprG*. Another possible explanation is that under *in vivo* stress, Δ*XprG/*Δ*PrtT* activates alternative virulence determinants that are not activated in the wild type. For example, we have previously shown that deletion of *prtT* resulted in the upregulation of four secondary metabolite clusters, including genes for the biosynthesis of toxic pseurotin A (Hagag et al., 2012). These factors could increase its virulence to wild type levels, despite its inability to produce secreted proteases. Functional gene redundancy could also account for our findings. Such redundancy has been repeatedly shown in *A. fumigatus*, following multiple deletions in large gene families encoding chitin synthases (Muszkieta et al., 2014), α-1,3-glucan synthases (Henry et al., 2012), and oligopeptide transporters (Hartmann et al., 2011).

Interestingly, the *A. fumigatus* strains generated here showed reductions in the expression of 21 of the 23 allergens identified in *A. fumigatus*, including those encoding the proteases Alp1/Aspf13, Alp2/Aspf18, Pep1/Aspf10, and Mep/Aspf5. Allergens encoded by fungal proteases activate an allergic Th-2 response *in vivo* by cleaving airway fibrinogen and generating fragments that act as TLR4 ligands on alveolar macrophages and airway epithelium (Millien et al., 2013). While secreted *A. fumigatus* proteases are not critical for infection of the immunocompromised host, our findings propose that they could be highly important in determining the response in fungal allergies. This possibility can be further explored with the *A. fumigatus* strains generated in this study.

## Author Contributions

ES, BH, SH, SA, YS, BF, and TK performed the experiments. AH, AB, OK, and NO conceived and planned the project and wrote the manuscript.

## Conflict of Interest Statement

The authors declare that the research was conducted in the absence of any commercial or financial relationships that could be construed as a potential conflict of interest.
