# Supplementary material for: Phenotypic and Proteomic Analysis of the Aspergillus fumigatus ΔPrtT, ΔXprG and ΔXprG/ΔPrtT Protease-Deficient Mutants
Source: Front Microbiol. 2017 Dec 12;8:2490. doi: 10.3389/fmicb.2017.02490 (PMC5732999; doi:10.3389/fmicb.2017.02490)
Supplement: Supplementary file 1 [file Data_Sheet_1.pdf]

Supplementary Data.

Construction and verification of the *A. fumigatus PrtT* disruption mutant. A- 5120-bp DNA fragment flanking the *A. fumigatus PrtT* gene (AFUA_4G10120) was generated by PCR, using the Expand high-fidelity PCR system (Roche Diagnostics), genomic DNA template from $AkuB^{KU80}$ and primers *PrtT* outer -5' and *PrtT* outer -3' designed to contain an *Asc*I restriction site at their 5' end (Supplementary Table S1). The product of this PCR was cloned into the TA vector pGEM-T-Easy (Promega). A 773-bp fragment, which included 307 bp of the N-terminal *PrtT* open reading frame, was then removed by digestion with *Xba*I and *Hind*III and replaced with a hygromycin-selectable marker to produce the p*PrtT*-D plasmid. The hygromycin cassette, containing 5' and 3' *Hind*III and *Xba*I restriction sites, respectively, was generated by PCR amplification using primers *Hyg* 5' and *Hyg* 3' (Table 3). For transformation, 10 µg of spin-purified *Asc*I-digested p*PrtT*-D plasmid was used. Transformation was performed as previously described by Romano et al (17). For Southern blot analysis, genomic DNA was extracted from the *A. fumigatus Ku80* strain and five independent transformants (Δ*PrtT-D1-5*). Southern hybridization analysis was performed as previously described (18). Briefly, 10 µg fungal genomic DNA samples were digested with *Xba*I and run on a 1% (w/v) agarose gel. The restricted DNA was transferred to a Nytran N nylon membrane (Schleicher & Schuell Bioscience, Keen, NH, USA) and hybridized with an $[\alpha\text{-}^{32}P]$dCTP- radiolabeled *PrtT* 5' probe at 65°C. The probe (785 bp) was generated by PCR with primers *PrtT* outer 5' and *PrtT* up 3' (Supplementary Table S1). Disruption of *PrtT* was verified by Southern blotting (Supplementary Fig. S1).

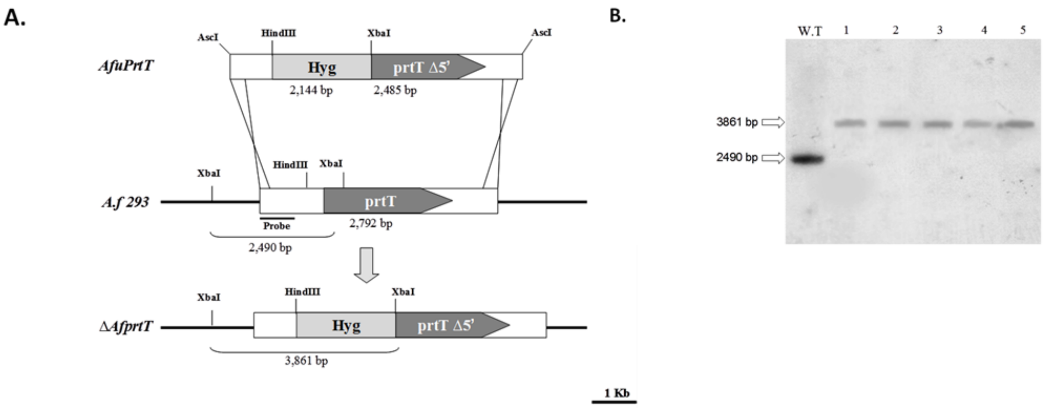

Supplementary Figure S1. Disruption of the *prtT* gene in *A. fumigatus*. (**A)** Schematic representation of the *prtT* WT locus and the *Asc*I-cut insert of plasmid pPrtT used for disruption. (**B)** Southern blot verification of the Δ*prtT*-D1-5 isolates and control *AkuB*^{KU80} strain. For the Southern blot analysis, genomic DNA (10 µg per well) was digested with *Xba*I, blotted, and hybridized with a ^{32}P-labeled *PrtT* 5'-flanking DNA probe, resulting in fragments of ~3.8 kb for the *PrtT* disrupted strains and ~2.5 kb for the *AkuB*^{KU80} control strain.

Construction and verification of the *A. fumigatus XprG* disruption mutant. A- 5750-bp DNA fragment flanking the *A. fumigatus xprG* homologe gene (AFUA_8G04050) was generated by PCR, using the Expand high-fidelity PCR system (Roche Diagnostics), genomic DNA template from *AkuB*^{KU80} and primers XprG up f and XprG D r designed to contain an *Asc*I restriction site at their 5' end (Supplementary Table S1).The product of this PCR was cloned into the TA vector pGEM-T-Easy (Promega). A 2945-bp fragment, which included the entire *xprG* open reading frame, was then removed by digestion with *Bgl*II and *Afl*II and replaced with a pyrithiamine -selectable marker to produce the pX*prG* -D plasmid. The pyrithiamine resistance cassette, containing 5' and 3' *Bgl2* and *Afl2* restriction sites, respectively, was generated by PCR amplification using primers PtrA Cassette f and PtrA Afl2 r and psk275 as template (19) (Supplementary Table S1). *Asc*I-digested pX*prG* -D plasmid was transformed into the *A. fumigatus AkuB*^{KU80} strain. Transformation was performed as previously described. For Southern blot analysis, genomic DNA was extracted from *AkuB*^{KU80} and two independent transformants (Δ*xprG-D1-2*). Southern hybridization analysis was performed as previously described (18). Briefly, 10 µg fungal genomic DNA samples were digested with *Bgl*II and run on a 1% (w/v) agarose gel. The restricted DNA was transferred to a Nytran N nylon membrane (Schleicher & Schuell Bioscience, Keen, NH, USA) and hybridized with an [α-^{32}P]dCTP- radio labeled *XprG* flanking region probe at 65°C. The probe (617 bp) was generated by PCR with primers Prob Bgl2 and Prob Bgl2-R2 (Supplementary Table S1). Disruption of *XprG* was verified by Southern blotting (Supplementary Fig. S2)

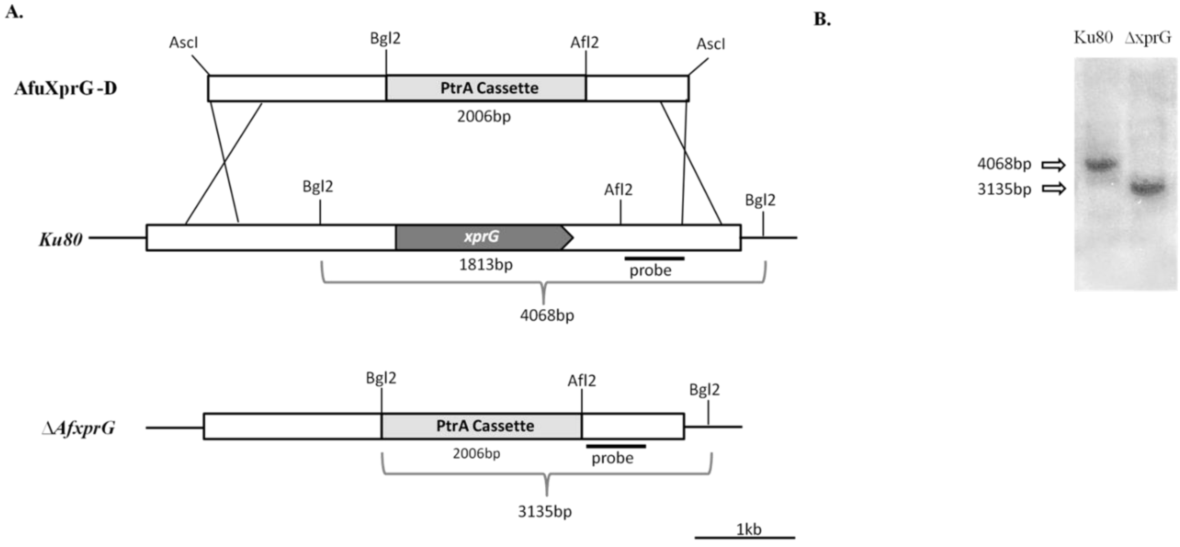

Supplementary Fig. S2. Deletion of the *xprG* gene in *A. fumigatus*. (**A)** Schematic representation of the *XprG* WT locus and the *Asc*I cut insert of plasmid pXprG-D used for disruption. (**B)** Southern blot verification of the Δ*XprG-D1* and control *AkuB*^{KU80} strain. For the Southern blot analysis, genomic DNA (10 µg per well) was digested with *Bgl*II, blotted, and hybridized with a ^{32}P-labeled DNA probe, resulting in fragments of for the 4068 bp for the *AkuB*^{KU80} strain and 3135 bp for the *XprG* disrupted strain.

Construction and verification of the *A. fumigatus PrtT*/*XprG* disrupted mutant. We created this mutant by transforming the plasmid used for the deletion of *PrtT* into the Δ*XprG-D1* mutant. After transformation, hygromycin-resistant transformants were purified and screened by PCR for putative insertion mutants. Mutation was further confirmed and verified for single integration of the transforming construct by Southern blotting (Supplementary Fig. S3).

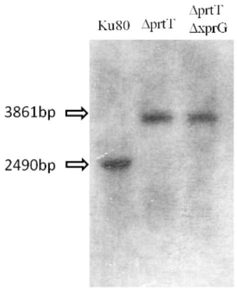

Supplementary Figure S3. Southern blot verification of the Δ*PrtT* /Δ*XprG* double mutant and control *AkuB*^{KU80} strain. For the Southern blot analysis, genomic DNA (10 µg per well) was digested with *Xba*I,

blotted, and hybridized with a ^{32}P-labeled *PrtT* 5’-flanking DNA probe, resulting in fragments of ~3.8 kb for the *PrtT*-disrupted strains and ~2.5 kb for the Ku80 control strain.

Construction and verification of the *A. fumigatus* complemented strains. The *PrtT-KI* (KI denotes knock-in or complemented) and Δ*XprG /PrtT* KI control strains were prepared by complementing the Δ*PrtT* and Δ*XprG /PrtT* strains with a circular plasmid containing the p*gpdA-phleomycin* cassette and the 5120-bp DNA fragment flanking (2 kb upstream and 0.4 kb downstream of the *PrtT* ORF) the above-described *A. fumigatus PrtT* gene. This plasmid (p*PrtT-KI)* was generated by cloning a p*gpdA-phleomycin* cassette into the p*PrtT*–D plasmid via an *Nde*I restriction site. The primers used to amplify the p*gpdA-phleomycin* cassette from pAN-8.1(20) were pAN-8.1 *Nde*I-5’ and pAN-8.1 *Nde*I-3’ (Supplementary Table S1). Verification of integration of pPrtT-D into the Δ*PrtT* and Δ*XprG /PrtT* strains was verified by colony PCR of transformants (Supplementary Fig. S4).

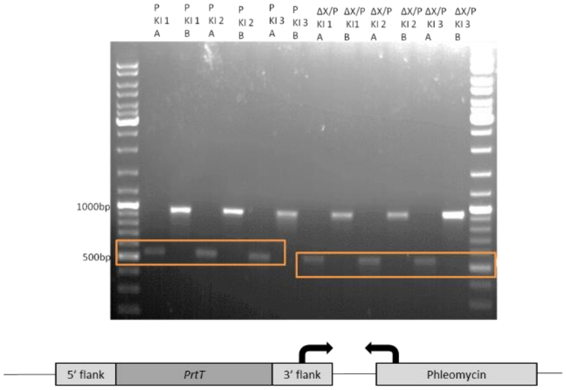

Supplementary Figure S4. PCR verification of integration of pPrtT-D into the Δ*PrtT* and Δ*XprG /ΔPrtT* strains to generate the *PrtT-KI* (PKI1-3)and Δ*XprG /PrtT* KI (ΔX/P KI1-3) rescued strains. Lanes A- PCR amplification with primers 5'PrtT and 3’PrtT validation to generate a 500 bp fragment (Supplementary Table S1). Lanes B-Control PCR performed using primers for Afu6g12750 (*RbdA*).

The *XprG-KI* control strain was prepared by complementing the Δ*XprG* strain with a circular plasmid containing the puc-*hph* cassete and a 3862 bp DNA fragment flanking the *A. fumigatus XprG* gene. The 3862 bp *XprG*-containing DNA fragment was cloned by PCR from *AkuB*^{KU80} genomic DNA using primers 5'HindXprG and 3'XbaXprG (Supplementary Table S1). Verification of integration of pXprG-D into the Δ*XprG* strain was verified by colony PCR of transformants (Supplementary Fig. S5).

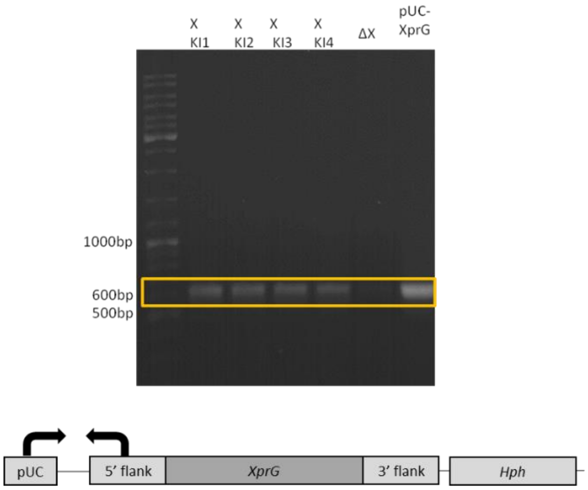

Supplementary Figure S5. PCR verification of integration of pXprG-D into the Δ*XprG* strain to generate the *XprG* KI (X KI1-4) rescued strains. PCR amplification with primers 5'XprG and 3'XprG validation to generate a 600 bp fragment (Supplementary Table S1). Controls- Δ*X* = Δ*XprG* strain, pUC XprG plasmid as template.

Proteomic analysis.

Cultures were harvested and filtered through Miracloth (Calbiochem). 10 % (w/v) trichloroacetic acid was added to the supernatant for 12 h at 4 °C in an ice-water bath with gentle shaking (15 rpm) to precipitate the secreted proteins. After centrifugation for 1h at 30000 × g, the pellets were emulsified in 0.5 ml supernatant and centrifuged at 14000 × g for 30 min. To remove detergents and other contaminants, the samples were treated with chloroform-methanol purification after Wessel-Flügge (1) and water-saturated ethyl acetate clean-up after Yeung and Stanley (2). The samples were vacuum-dried for approximately 1-5 min to almost dryness. Proteins were solubilised in 200 µl ammonium bicarbonate (50 mM) / 2,2,2-trifluoroethanol 1:1 (w/v) and sonicated in a water-bath sonicator for 10 min. Sample were centrifuged for 20 min at 20,000 × g, 20 °C. Supernatant was transferred in a new 1.5 ml microcentrifuge tube. Protein concentration was detected using Millipore DirectDetect and 70 mg of samples were denaturated at 90 °C, 500 rpm for 10 min. Disulphide bonds were reduced with 5 µl of 100 mM tris(2-carboxyethyl)phosphine and reduced cysteine residues were alkylated with 5 µl 500 mM iodoacetamide for 30 min at room temperature, 500 rpm. Samples were evaporated until a final volume of about 5 µl was left and diluted in 100 µl 50 mM ammonium bicarbonate. Proteins were digested over night with trypsin in a ratio of 1:25 trypsin to protein at 37 °C, 400 rpm. Samples were labelled with iTRAQ isobaric labelling for quantitative proteomics

approach to analyse the protein ratios between the knock-out mutants and the wild type. The iTRAQ labelling was conducted according to the manufacturer's instructions (Applied Biosystems). The labeling reactions were incubated for 1 h at room temperature, subsequently quenched with water, combined and further fractionated on a strong cation exchanger (Thermo Fischer HyperSep SCX SPE 50 mg/ml) in five different fractions. The initial equilibrium buffer for the SCX fractioning was 25 % ACN v/v, 0.05 % HCOOH v/v and 0 mM ammonium bicarbonate, the four following fractions were eluted from the SCX column with 25mM, 50 mM, 100 mM and 400 mM ammonium bicarbonate solutions. The last fraction (400 mM ammonium bicarbonate) and the flow-through (0 mM ammonium bicarbonate, 25 % ACN v/v, 0.05 % HCOOH v/v) were combined. The volume of the fractions were reduced in a vacuum centrifuge, peptides were diluted in 20 µl of 0.05 % TFA v/v in 2/98 v/v ACN/H_2O (0,05 % TFA in 98/2 H2O/ACN) and sonicated in a water bath sonicator for 15 min. An Ultimate 3000 nano RSLC system coupled to a QExactive Plus mass spectrometer (both Thermo Scientific Fischer) was used as a mass analyser. 4 µl sample was injected for each analysis and per biological replicate, three analytical replicates were applied. For enrichment and desalting, an online pre-concentration set-up was used with a nano trap column (Acclaim PepMap 100, 2 cm x 75 µm, 3 µm) at a flow rate of 5 µl / min for 4 min. The pre-concentrated sample was eluted on the analytical column (Acclaim PepMap RSLC, 50 cm x 75 µm, 2 µm). The binary mobile phase consisted of ((A) 0.1% v/v formic acid in H_2O and (B) 0.1% v/v formic acid in 90/10 ACN/H_2O) to separate the peptides with a non-linear 135 min gradient elution at 40°C: 0-5 min at 4% B, 10 min at 6.5% B, 15 min at 7.5% B, 20 min at 8% B, 25 min at 8.6% B, 30 min at 9.2% B, 35 min at 9.9% B, 40 min at 10.6% B, 45 min at 11.3% B, 50 min at 12.2% B, 55 min at 13.4% B, 60 min at 14.9% B, 65 min at 17% B, 70 min at 19.1% B, 94 min at 40% B, 199 min at 52% B, 103 min at 68% B, 106-114 min at 96% B, and 115-135 min at 4% B. . Positive charged ions were generated at the Nanospray Flex Ion Source (Thermo Fisher Scientific) with a stainless steel emitter at 2.2 kV spray voltage. The capillary temperature was set to 220 °C, the S-lens RV level to 50. The calibration was carried out with the positive CalMix solution (external calibration) and with the lock mass of m/z 445.12003 amu for $[C_2H_6SiO]_6$ (polydimethylsiloxane) (internal calibration). In turns with the samples, Cytochrome C and blind injections were used to monitor the performance of the LC during the whole analysis. Further on, the UV absorption at 214 nm wavelength was recorded. The mass range of the precursor ions was set to m/z 300 – 1600 at a

resolution of 70k full width at half maximum (FWHM) with a maximum injection time of 120 ms and an automatic gain control target of 1 x 10^6. (The quadrupole / orbitrap mass analyser worked in Full MS/ddMS^2 (TopN) mode.) For data-dependent acquisition, up to eight most abundant precursor ions with an assigned charge of z = 2-6 for further fragmentation, selected by the quadrupole in each scan cycle with an isolation width of m/z 2.0. In the collision cell, the fragments were generated at a normalised collision energy of 36 V with nitrogen gas. Precursor ions were excluded dynamically for 35 s. With a maximum injection time of 120 ms and an automated gain control target of 2 x 10^5, the fragment ion resolution was set to 17.5k FWHM. The software Thermo/Dionex Chromeleon Xpress v6.80 SR13 build 3818 and the Thermo QExactive Plus Tune/Xcalibur v3.0.63 2.3 build 1765 graphical interface software was used on the LC-MS/MS instrument. The raw files generated by the LC-MS/MS were further processed by the software Proteome Discoverer v1.4.0.288 (Thermo). Tandem mass spectra were searched against the AspGD *Aspergillus fumigatus Af293* (www.aspergillusgenome.org/download/sequence/A_fumigatus_Af293/current/A_fumigatus_Af293_current_orf_trans_all.fasta.gz; status 2015/07/05) protein database with the algorithm of MASCOT v2.4.1 (Matrix Science, UK), SEQUEST HT, and MS Amanda for database analysis with a maximum of two missed cleavages. The mass tolerances were set to 10 ppm. for the precursor ion and to 0.02 Da as a fragment mass tolerance. As modifications, oxidation of Met (dynamic), 4-plex peptide iTRAQ labeling of the N-termini of peptides (dynamic), such as of the side chains of lysine (dynamic) and carbamidomethylation of Cys (static, due to usage of idoacetamide) were considered. iTRAQ reporter ion ratios were calculated by comparison of all biological replicates of the single (ΔprtT / WT and ΔxprG / WT) or double (ΔprtTΔxprG / WT) mutant strains against the wild type (115/114, 116/114, 117/114, 116/115, 117/115, 114/115, 117/116, 114/116, 115/116). For validation (q value) of the peptide spectra matches, a reverse decoy database and a percolator node were used with a maximum ΔCn of 0.05 and a target false discovery rate of 0.01 (strict) and 0.05 (relaxed). A minimum of 2 peptides per protein hit were required and only unique peptides were used for the quantification. A regulation ≥1.5-fold was considered as significant threshold for up- or down-regulations. Calculated ratios that are based on 2-4 reporter ions (average) and an average variability 30 - 50% were considered as medium confidence, ≥4 reporter ions (average) and ≤30% were considered as high confident data. The deletion of the transcription factors XprG

and PrtT led to a strong reduction in the amount of secreted proteins. Due to this reduced levels of secreted proteins in the deletion mutant strains, up to three replicates were required and were pooled to obtain the minimal total protein amount of 70 µg for LC-MS/MS analysis. In order to take into consideration the significant reduction of protein secretion, the, protein ratios were afterwards normalized to the protein concentration of secreted proteins determined in the medium. To determine these normalizaiton factors, the protein concentrations measured in the culture supernatant were divided by the protein concentration in the culture supernatant of the wild type strain.

Histological analysis of lung sections following intranasal murine infection with *A. fumigatus* conidia. Six-week-old immunocompetent female ICR mice were inoculated intranasally with 1×10^7 conidia and sacrificed after 24 h or 48 h. Histological analysis was performed with gomori methenamine silver stain (GMS, stains fungal elements black) or haematoxylin and eosin stain (H&E, stains host-cell nuclei purple, cytosol pink).

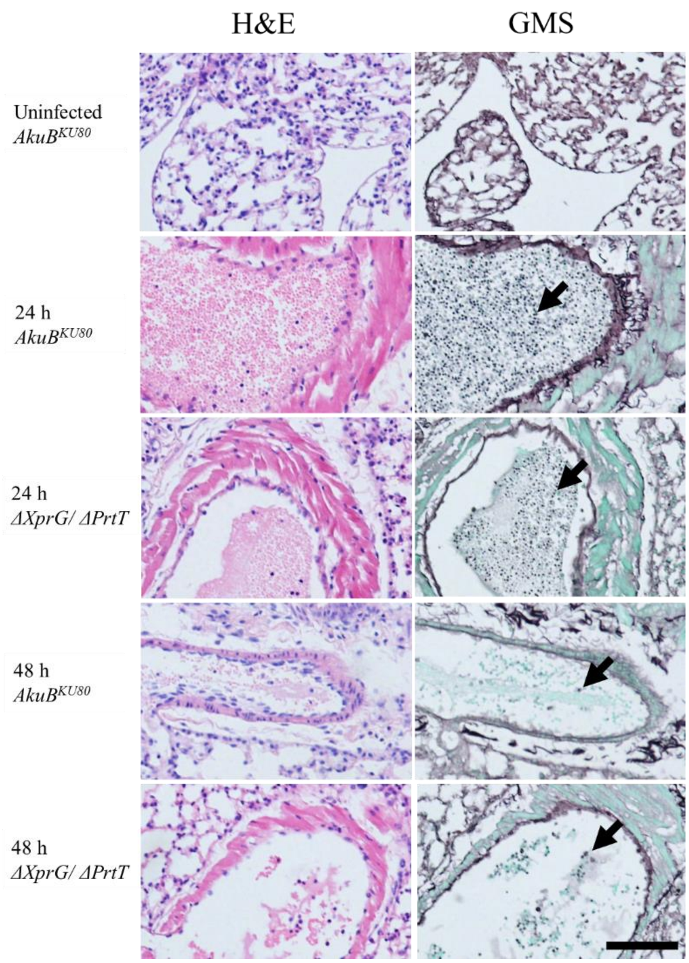

Supplementary Figure S6. Immunocompetent mice mount a similar immune response to *ΔXprG/ΔPrtT* and control *AkuB*^{KU80} pulmonary infection. 6-week old female ICR mice were infected intranasally with 10^7 *ΔXprG/ΔPrtT* or control *AkuB*^{KU80} conidia. The mice (*n* = 5 each group) were sacrificed 24 h or 48 h following infection. Their lungs were surgically excised and histological sections were stained with GMS (stains fungi black, right panel, black arrow) or H&E (stains host cell nuclei purple, cytosol in pink, left panel). Size-bar = 100 µm.

References

1. Wessel D, Flügge UI. A method for the quantitative recovery of protein in dilute solution in the presence of detergents and lipids. Anal Biochem. 1984 138:141-3.

2. Yeung YG, Stanley ER. Rapid detergent removal from peptide samples with ethyl acetate for mass spectrometry analysis. Curr Protoc Protein Sci. 2010. Chapter 16: Unit 16.12.

Supplementary Table S1: Primers used in this study

Oligonucleotide	Sequence 5’→3’
XprG up f	ATGGCGCGCCCATGCATACGTTAGGTGGTT
XprG D r	ATGGCGCGCCACTCTGTTGCCACGTACCA
PtrA Cassette f	TAAGATCTAGGCCAATTGATTACGGGAT
PtrA Afl2 r	ATCTTAAGCCTAGATGGCCTCTTGCAT
Prob Bgl2	AGTAATGACTCGTGGCAGCT
Prob Bgl2-R2	TGAACCCTCGAGCTTACCGA
PrtT outer 5'	ATGGCGCGCCGCACTGATCTCGTCCTATCGGTT
PrtT outer 3'	ATGGCGCGCCCTAAAGTCTCCTACCTACCTCAG
Hyg 5'	ATAAGCTTGTCGACAGAAGATGATAT
Hyg 3'	ATAAGCTTGCTCTCCCTTATGCGACTCCTGCA
PrtT up 3’	AGTGACAGAACAGGCGACAG
pAN-8.1 NdeI 5’	ATCATATGGAATTCCCTTGTATCTCTACACAC
pAN-8.1 NdeI 3’	TGCACCATATGCGGTGTGAAATA
gpdA 5’	TCTCCAACGTTCTTGCACC
gpdA 3’	CCACTCGTTGTCGTACCAGG
prtT inner 5’	GGTGATAGCGAAACAGAAGAGACAAG
prtT inner 3’	ATAGACTCCACTGGTAGTTAGGTGTTCTG
5'HindXprG	TAAAGCTTCAGCCTACAGACTGTCTTTCTC
3'XbaXprG	TATCTAGAGCAGAGGGACTGGGATATTTG
5'PrtT validation	GTAACGACTGAGGTAGGTAGGA
3'PrtT validation	TTTAAGGCTGCCAACAGCT
5'XprG validation	CAATGGCTATCGGCTCTCTTT
3'XprG validation	TGGTTAGCGTCTGACTTTG

Supplementary Table S3

Category	Sub-Category	Gene number/Name	*ΔPrtT*	*ΔXprG*	*ΔXprG/PrtT*
**Proteases**	Serine Proteases	*DppV/Afu2g09030*	NS*	0.51†	0.29
**(n=24)**		*DppIV/Afu4g09320*	0.63	0.42	0.3
		Cp6/Afu5g01200	0.47	0.45	0.18
		Sxa2/Afu2g03510	0.44	0.37	0.24
		DapB/Afu3g07850	0.42	0.64	0.28
		Cp3/Afu6g13540	0.32	0.51	0.29
		Cp1/Afu5g07330	0.32	0.53	0.27
		Afu2g01250	0.26	0.48	0.32
		AorB/Afu6g10250	0.52	0.57	0.36
		KexB/Afu4g12970	0.45	0.56	0.31
		SedC/Afu3g08930	0.41	0.32	0.36
		Sed/Afu4g03490	0.32	0.42	0.25
		Alp1/Aspf13/Afu4g11800	0.31	NS	0.34
		Alp2/Aspf18/Afu5g09210	0.31	NS	0.29
	Metalloproteases	*Afu1g05960*	0.46	0.59	0.29
		Ape3/Afu2g00220	0.4	NS	0.35
		Cps1/Afu3g07040	0.36	0.6	0.32
		Afu6g06800	0.34	0.47	0.35
		Lap1/Afu4g04210	0.32	0.32	0.25
		Lap2/Afu3g00650	0.27	NS	0.24
		Mep/Aspf5/Afu8g07080	0.39	0.3	0.36
	Aspartic Proteases	*CtsD/Afu4g07040*	0.46	NS	0.4
		Pep1/Aspf10/Afu5g13300	NS	0.46	0.29
		Pep2/Afu3g11400 1	0.35	0.64	0.31
**Glucanases**					
**(n=18)**		*Eng6/Afu6g14540*	NS	0.42	0.32
		BtgE/Afu8g05610	NS	NS	0.55
		Exg6/Afu6g13270	0.54	0.62	0.37
		Afu2g03980	0.53	NS	0.31
		Utr2/Afu2g03120	0.51	NS	0.37
		Neg1/Afu8g07120	0.48	0.59	0.29
		Eng7/Afu3g03080	0.48	NS	0.32
		Afu7g06150	0.4	0.53	0.29
		Egl2/Afu6g11600	0.4	0.65	0.35
		Engl1/Afu1g04260	0.35	NS	0.31
		Afu3g03870	0.34	0.35	0.34
		Afu8g06830	0.33	NS	0.35
		ExgO/Afu1g14450	0.32	NS	0.29
		Afu6g03230	0.31	0.52	0.38

		Bgt3/Afu5g08780	0.31	0.51	0.3
		Aspf9/Afu1g16190	0.26	0.52	0.26
		Bgt2/Afu3g00270	0.25	NS	0.31
		Afu1g12560	0.23	0.33	0.32
**Chitinases**					
**(n=6)**		*Afu3g07160*	0.46	0.42	0.42
		ChiB1/Afu8g01410	0.41	NS	0.36
		Chi3/Afu7g05140	0.37	NS	0.33
		Afu3g11280	0.36	NS	0.33
		Afu6g09307	0.33	0.64	0.32
		ChiA1/Afu5g03760	0.32	0.6	0.3
**Allergens**					
**(n=17)**		*Aspf4/Afu2g03830*	NS	NS	0.65
		Aspf28/Afu6g10300	NS	0.51	0.51
		Aspf12/Afu5g04170	0.6	0.48	NS
		Aspf11/Afu2g03720	0.59	0.56	0.45
		Aspf3/Afu6g02280	0.55	NS	0.48
		Aspf27/Afu3g07430	0.54	NS	0.42
		Aspf29/Afu5g11320	0.51	0.46	0.5
		Aspf7/Afu4g06670	0.48	NS	0.35
		Aspf6/Afu1g14550	0.45	0.35	0.63
		Aspf23/Afu2g11850	0.41	0.53	0.24
		Aspf1/Afu5g02330	0.4	0.48	0.35
		Aspf2/Afu4g09580	0.39	0.41	0.29
		Aspf22/Afu6g06770	0.38	0.49	0.52
		Aspf13/Afu2g12630	0.34	0.38	0.39
		Aspf8/Afu2g10100	0.3	0.31	0.34
		Aspf34/Afu3g03060	0.28	NS	0.3
		Aspf17/Afu4g08960	0.28	NS	0.26

NS-not significant

† Fold change vs. *AkuB*^{KU80} wild-type strain
